# Genipin cross-linked decellularized tracheal tubular matrix for tracheal tissue engineering applications

**DOI:** 10.1038/srep24429

**Published:** 2016-04-15

**Authors:** Fei Sun, Yuan Jiang, Yanfei Xu, Hongcan Shi, Siquan Zhang, Xingchen Liu, Shu Pan, Gang Ye, Weidong Zhang, Fangbiao Zhang, Chonghao Zhong

**Affiliations:** 1 Department of Cardiothoracic Surgery, College of Clinical Medicine, Yangzhou University, Yangzhou, 225001, Jiangsu Province, China; 2 Yangzhou University Medical College, Yangzhou, 225001, Jiangsu Province, China; 3 Jiangsu Key Laboratory of Integrated Traditional Chinese and Western Medicine for Prevention and Treatment of Senile Diseases, Yangzhou, 225001, Jiangsu Province, China

## Abstract

Decellularization techniques have been widely used as an alternative strategy for organ reconstruction. This study investigated the mechanical, pro-angiogenic and *in vivo* biocompatibility properties of decellularized airway matrices cross-linked with genipin. New Zealand rabbit tracheae were decellularized and cross-linked with genipin, a naturally derived agent. The results demonstrated that, a significant (p < 0.05) increase in the secant modulus was computed for the cross-linked tracheae, compared to the decellularized samples. Angiogenic assays demonstrated that decellularized tracheal scaffolds and cross-linked tracheae treated with 1% genipin induce strong *in vivo* angiogenic responses (CAM analysis). Seven, 15 and 30 days after implantation, decreased (p < 0.01) inflammatory reactions were observed in the xenograft models for the genipin cross-linked tracheae matrices compared with control tracheae, and no increase in the IgM or IgG content was observed in rats. In conclusion, treatment with genipin improves the mechanical properties of decellularized airway matrices without altering the pro-angiogenic properties or eliciting an *in vivo* inflammatory response.

In June 2008, the world’s first fully tissue-engineered tracheal transplantation with a non-immunogenic decellularized human donor trachea reseeded with bone-marrow-derived mesenchymal stem cells (MSCs) and respiratory cells was successfully transplanted into a 31-year-old woman^1^. However, complications arose a few months later when the patient’s airway narrowed again and collapsed, requiring doctors to insert a stent to keep her airway open. The doctors removed it 6 months later, but the patient had to have several more stents inserted and removed in the past 5 years^2^. Finally, the tissue-engineered trachea remained open over its entire length, was well-vascularized, was completely re-cellularized with respiratory epithelium, and had normal ciliary function and mucus clearance, suggesting no significant loss of airway nerve function 5 years after transplantation^3^. Tissue engineering is an emerging interdisciplinary field of research, and clinical applications focus on the repair, replacement or regeneration of cells, tissues or organs to restore impaired function. The application of scaffolds or matrices to grow new tissues or organs from isolated cells, tissue or synthetic compounds is the basic principle of tissue engineering. In this manner, immunosuppression is avoided^4^.

When engineering the trachea, the scaffold should maximally mimic the target tissue characteristics, such as the anatomical structure and mechanical properties. The ideal tracheal scaffold will need to be processed to remove antigenicity to avoid immunorejection while maintaining extracellular matrix integrity to provide sufficient mechanical strength to maintain airway ventilation^5^. One method used to achieve this goal is enzymatic decellularization, which aims to eliminate antigenic materials while preserving the extracellular matrix of the source tissue^6^. This method provides a nearly nonimmunogenic acellular airway matrix with fairly native mechanical properties. Moreover, the decellularized matrix continues to retain proangiogenic factors, such as basic fibroblast growth factor (bFGF), which can aid in promoting the formation of new blood vessels^7^. In other words, the decellularized scaffold appears to exhibit the properties of an “ideal matrix”, namely biocompatibility, bioabsorbability, nonimmunogenity, support of cell attachment and growth, and an ability to induce angiogenesis^4^. To date, decellularized scaffolds have been widely used for animal transplantation experiments^8^ as well as in some clinical practices^9^. Baiguera and colleagues^10^ demonstrated that stored human decellularized tracheae were increasingly degraded, resulting in a loss of the extracellular matrix architecture, particularly the collagenous and elastic fibre structure, as well as a decrease in mechanical and angiogenic properties. Partington and associates^11^ found that substantial decreases in the glycosaminoglycan (GAG) content in tracheal cartilage were evident throughout the decellularization process, which could reflect a change in the tissue structure that may manifest as biomechanical instability *in vivo*. Here, genipin is considered to be a naturally derived cross-linking agent that improves the mechanical properties of decellularized tracheae. Haag and colleagues^12^ demonstrated that treatment with genipin improved the mechanical properties, without altering the pro-angiogenic properties of the decellularized airway matrix or eliciting an *in vivo* inflammatory response.

The current study aimed to investigate the effects of a natural cross-linking agent (genipin) on the mechanical, pro-angiogenic and *in vivo* biocompatibility properties of decellularized airway matrices.

## Materials and Methods

### Ethics statement

This study was carried out in strict accordance with the recommendations in the Guide for the Care and Use of Laboratory Animals of the National Institutes of Health. All protocol were performed in accordance with the approved guidelines and were approved by the Ethics Committee of the Medical College of Yangzhou University.

### Animals

New Zealand white rabbits (n = 32), weighing 2.5 ± 0.27 kg, were used as trachea donors. Forty-five male SD rats weighing 302 ± 29 g acted as recipients.

### Study design

Whole tracheae were harvested from 32 donor rabbits. Eight of the tracheae were used to verify the effectiveness of the decellularization (Movat, 4′-6-diamidino-2-phenylindole, glycosaminoglycan and type II collagen staining), and pro-angiogenic and mechanical evaluation. Other tracheae (n = 12) were decellularized, treated with genipin (1%) and glutaraldehyde (0.625%), and subjected to morphological (hematoxylin-eosin, Masson trichrome staining and scanning electron microscopy), pro-angiogenic and mechanical property evaluations. Nine decellularized tracheae were cut into three pieces, yielding a total of 27 pieces (approximately 1.5 cm long): 9 were used as a decellularized group, 9 were cross-linked with genipin, and the other 9 were cross-linked with glutaraldehyde. The last 3 native tracheae were also cut into three pieces, and all of the pieces were xenotransplanted into SD rats (n = 36), with 9 other SD rats used as a sham group. Biopsy specimens and blood samples were taken from recipient rats 7, 15 and 30 days after implantation right before being euthanized.

### Trachea harvesting

Tracheae were harvested in a sterile manner, using standard surgical procedures, as previously reported^13^. Briefly, a midline sternotomy was performed to expose the entire trachea from the larynx to the origin of the main bronchi. The trachea was then separated just below the main carina and gently isolated from the oesophagus and mediastinal tissue, cut below the larynx and subsequently stored in cold PBS containing 1% antibiotic and antimycotic solution.

### Decellularization process

Tracheae were decellularized with seven cycles of a detergent–enzymatic treatment protocol as previously described^13^; namely, the tracheae were incubated in sterile filtered water for 48 h at 4 °C in 4% sodium deoxycholate (Sigma) diluted in distilled water and rotated continuously for 4 h at 37 °C, and in DNase-I (2 kU mL^−1^; Sigma) in 1 mol/L NaCl (Sigma) with continuous rotation for 3 h at 37 °C. After the washing steps, the trachea samples were stored in PBS containing 1% antibiotic and antimycotic solution at 4 °C overnight; the following day, the next cycle was initiated. To determine the decellularization efficacy, a Movat pentachrome stain kit (Leagene, Beijing, China) was employed to evaluate the connective tissues, including the cartilage, elastic fibres, collagen, reticular fibres, and muscle. Sections were also stained with 4′-6-diamidino-2-phenylindole (DAPI; KeyGEN, Nanjing, China) to detect nuclear material. Safranin O staining (Solarbio, Beijing, China) was performed to evaluate the glycosaminoglycan expression. An immunohistochemical analysis of type II collagen (antibodies all from ABM, Carlsbad, USA) was performed to evaluate the type II collagen expression.

### Cross-linking treatment

The decellularized tracheae were cross-linked in 1% w/v aqueous genipin (Merlin, Hangkong, China) or 0.625% w/v aqueous glutaraldehyde (Aladdin, Shanghai, China) solution buffered with PBS for 1 h at 37 °C under constant agitation. The treated tissues were then thoroughly washed with sterile PBS (at least three changes) containing 1% antibiotic and antimycotic solution, with each washing cycle lasting for 10 min. The final tissues were stored in PBS containing 1% antibiotic and antimycotic solution at 4 °C.

### Histological analysis

Parts of the trachea samples (native, decellularized, and genipin-treated) were fixed for 24 h in 10% neutral buffered formalin solution in PBS (pH 7.4) at room temperature. They were washed in distilled water, dehydrated in graded alcohol, embedded in paraffin, and sectioned at a 5 mm thickness. Adjacent sections were stained with hematoxylin and eosin (H&E) stain (Leagene, Beijing, China) and Masson trichrome stain (Leagene, Beijing, China) to evaluate the tissue morphology.

### Scanning electron microscopy

To qualitatively evaluate the decellularized matrix structure, tracheal (native, decellularized, and genipin treated) matrices (n = 2 for each condition) were fixed with 2.5% (v/v) glutaraldehyde in a buffered solution of 0.1 M sodium cacodylate buffer (pH 7.2). After rinsing in cacodylate buffer, the specimens were dehydrated through an ethanol gradient, critical point dried, sputter coated with gold and observed via scanning electron microscopy (SEM; S-4800, Hitachi High-Technologies Corporation, Tokyo, Japan).

### Biomechanical tests

The mechanical properties of the tracheal (native, decellularized, genipin treated and glutaraldehyde treated) matrices (n = 3 for each condition) were evaluated via uniaxial tensile tests (Instron 3367, Canton, USA). The tissues were subjected to increasing uniaxial tensile stresses until rupture, as confirmed by the loss of load and the appearance of tears in the tissue. The specimens were clamped into sample holders, a pre-load (preliminary force) of 1 N was applied and the testing was initiated at a constant elongation rate of 30 mm/min at room temperature. The tensile tester recorded the load and elongation of the tissue in real time. Parameters such as the maximum force (N) and elastic modulus (MPa) were recorded.

A PerkinElmer DMA Q800 instrument (TA Instruments, Inc, New Castle, USA) was used to assess and record the compressive properties of the tracheal (native, decellularized, genipin-treated and glutaraldehyde-treated) samples (n = 3 for each condition) in real time. For this testing, the trachea specimens (5 mm diameter) were prepared with the adventitia and lamina propria removed. The exact measurements were input into the software, and the specimen was appropriately positioned in the instrument. A force was then applied at 200 mN/min up to 3,000 mN, and the Young’s (compressive) modulus (MPa) ± SD was determined for each analysis.

### *in vivo* pro-angiogenic properties

Chicken embryo chorioallantoic membrane (CAM) assays were used as an *in vivo* model to evaluate the angiogenic properties of fresh, decellularized, genipin-treated and glutaraldehyde treated tracheal grafts. As previously reported^14^, fertilized chicken eggs (n = 3 for each condition) were incubated under constant humidity at 37 °C. On incubation day 3, a square window was opened in the shell to detach the developing CAM after the removal of 2 mL of albumin. The window was sealed with a glass, and the eggs were returned to the incubator. On day 8 of incubation, 1 mm^3^ tracheal samples were placed on the CAM. Next, 1 mm^3^ sterilized gelatine sponges (Johnson & Johnson, New Jersey, USA) containing vehicle alone (PBS) were used as negative controls. All procedures were performed under sterile conditions. CAMs were examined daily until day 12 and photographed in ovo. The angiogenic response was evaluated as the number of vessels converging toward the implants and the sponges on day 12.

### Matrix implantation and postoperative observation

Matrix samples (1.5 cm) were implanted under the dorsal skin of SD rats (n = 45). Rats were randomly divided into 5 experimental groups (nine animals each): Group A were sham rats; Group B rats received the native tracheal matrix; Group C rats received the decellularized tracheal matrix; Group D rats received the genipin (1%)-treated tracheal matrix; and Group E rats received the glutaraldehyde (0.625%)-treated tracheal matrix. Rats were killed (three animals for each group) on days 7, 15 and 30. Blood samples were taken, and the implanted tracheae were analysed both macroscopically and stained with H&E.

### Analysis of tissue from rat recipients

Blood samples from rats were analysed to identify the levels of total circulating IgG and IgM (mg/ml) with an enzyme-linked immunosorbent assay kit (Bio-Swamp, Beijing, China). Tissue samples were immersed in 10% buffered formalin for 24 h and embedded in paraffin. Then, sections were stained with H&E and examined for the presence of inflammatory cells.

### Statistical analysis

All quantitative data are expressed as the mean ± SD. Differences between groups were assessed using the one-way analysis of variance with the post hoc t-test using a commercial statistical package (SPSS 19.0, Inc. Chicago, IL). Differences were considered significant at the 95% level (p < 0.05).

## Results

### Matrix characterization

**Figure 1:**
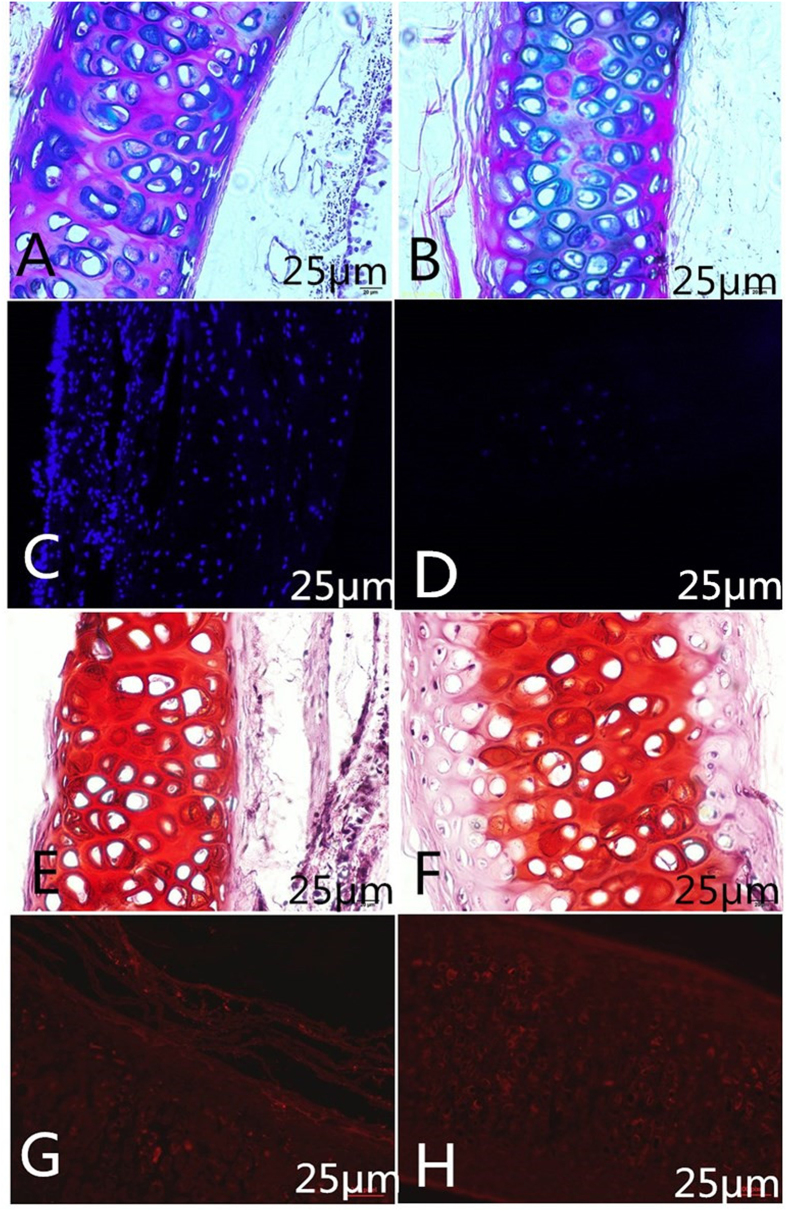
Characterization of decellularized rabbit tracheal matrix. Movat pentachrome staining showed that compared with the native trachea (**A**), after seven decellularization cycles (**B**), the three-dimensional architecture and composition of the matrix remained intact and was virtually unaltered. 4′-6-Diamidino-2-phenylindole staining of the native trachea (**C**). After seven detergent-enzymatic treatment cycles (**D**), the nuclei were completely removed in the noncartilaginous tissue, and a small number of nuclei remained only in the thick cartilage and cell debris retained in the lacunae. Glycosaminoglycan expression visualized by safranin O staining in native trachea (**E**) and trachea after seven detergent-enzymatic treatment cycles (**F**); glycosaminoglycan was stained red/orange. The immunolabelling of type II collagen within the cartilage matrix suggested that compared with the native trachea (**G**), the trachea after seven detergent-enzymatic treatment cycles (**H**) experienced no significant decline in collagen content. The scale bar is 25 μm.

The decellularized tracheal matrix maintained a sufficient extracellular matrix and structural rigidity. Movat’s pentachrome staining (Fig. 1A,B) showed that the histological structure of the native rabbit trachea included a ciliated epithelium covering the basal membrane, cartilage, and muscular tissue. After 7 decellularization cycles, the three-dimensional architecture and composition of the matrix remained intact and was virtually unaltered, including the preservation of the basement membrane. Nuclei stained with 4′-6-diamidino-2-phenylindole (Fig. 1C,D) were completely removed in the noncartilaginous tissue, and a small number of nuclei remained only in the thick cartilage and cell debris retained in the lacunae after seven cycle. Safranin O staining (Fig. 1E,F) showed that glycosaminoglycan expression was mainly concentrated in the cartilaginous region. After the seventh cycle, there was a significant loss of glycosaminoglycan in the outer layer of the tracheal cartilages, with the remaining glycosaminoglycan mainly expressed in the core area of the cartilage. Immunolabelling of the type II collagen (Fig. 1G,H) within the cartilage matrix suggested that, compared with the native trachea, the decellularized tracheal matrix experienced no significant decline in the collagen content.

### Genipin-treated tracheae

**Figure 2:**
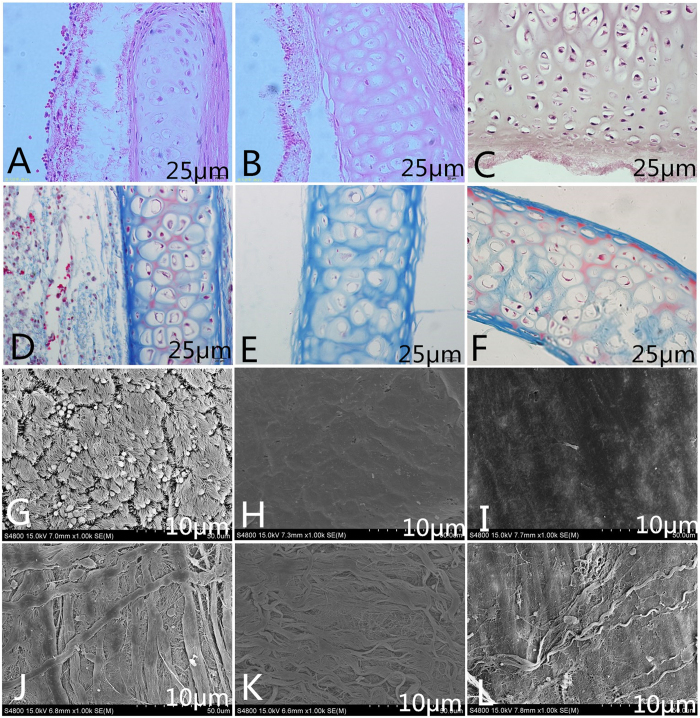
Characterization of genipin-treated decellularized tracheal matrix. Hematoxylin and eosin (**A**–**C**), Masson trichrome staining (**D**–**F**) and SEM evaluation luminal (**G**–**I**) and external surfaces (**J**–**L**) of native trachea (**A**,**D**,**G**,**J**), trachea after seven detergent-enzymatic treatment cycles (**B**,**E**,**H**,**K**), and decellularized tracheal matrix cross-linked using 1% genipin solution for 1 h (**C**,**F**,**I**,**L**). The total network of ECM fibres appeared compact, including the network of collagen, reticular and elastic fibres, which resulted in a more organized and less fragmented structure than in decellularized samples. The collagen was stained bluevia Masson trichrome staining. SEM showsirregular collagen fibres characterizing the external surface, whereas the basal lamina was maintained on the luminal surface. The scale bars are 25 μm in A-F and are 10 μm in panels (**G**–**L**).

After fixation, the genipin-treated decellularized tracheal matrices were stiffer than the non-genipin treated decellularized tissues. Histological examinations (H&E and Masson trichrome staining) showed a nearly intact ECM framework, which appeared as a denser and more compact structure compared to that of the decellularized tracheal matrices. The cartilage, basement membrane and submucosa of the genipin-treated decellularized tracheal matrices (Fig. 2C,F) were more organized and less fragmented than those of the decellularized samples (Fig. 2B,E). These results were also confirmed by SEM analysis (Fig. 2G–L).

### Biomechanical properties

Uniaxial tensile tests were carried out on the decellularized tracheal matrices, showing that the strain at failure (the amount of force required to break the trachea) was similar for the fresh tracheae. Although the ultimate tensile strength did not significantly influence the strain capacity of the tracheal matrices after decellularization, the compressive properties significantly decreased in the decellularized trachea compared with the native tracheal matrices. Genipin (11.19 ± 0.93N) and glutaraldehyde (10.83 ± 0.92N) cross-linked samples showed an improved mechanical response, as an average trend, with comparable strain at break values for all of the investigated cases compared to the decellularized tracheal matrices (8.13 ± 0.49N). Similarly, the application of compressive forces of up to 3,000 mN revealed that genipin (2.06 ± 0.18 MPa) or glutaraldehyde (2.11 ± 0.21 MPa) cross-linking treatment resulted in an increase in the compressive properties of the matrices compared to that of decellularized tracheal matrices (1.43 ± 0.12 MPa). (Table 1).

### *in vivo* pro-angiogenic properties

**Figure 3:**
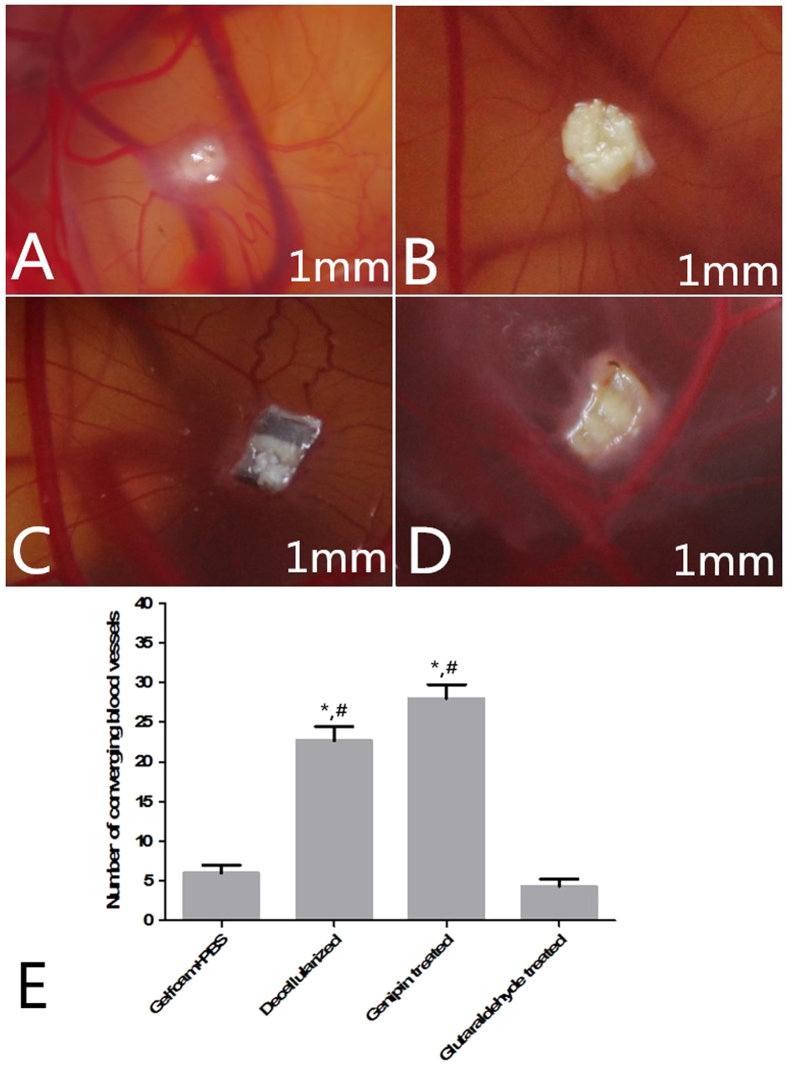
*In vivo* pro-angiogenic properties of Gelfoam (**A**), decellularized (**B**), genipin-treated (**C**), and glutaraldehyde-treated tracheal matrices (**D**). Representative examples of chicken chorioallantoic membrane (CAM) implanted with fragments of Gelfoam, decellularized, genipin-treated and glutaraldehyde-treated tracheal matrices. Samples were placed on the CAM surface of 8-day-old embryos and photographed 4 days later. The samples induced a “spoke-wheel” pattern of new vessels. The scale bar is 1 mm. (**E**) Effect of Gelfoam, decellularized, genipin-treated and glutaraldehyde-treated tracheal matrices on the number of converging blood vessels 4 days post-implantation. Genipin cross-linking induces a significant (p < 0.05) increase, comparable to the control, in the number of newlybuilt blood vessels. *p < 0.05 with respect to negative control; ^#^p < 0.05 with respect to glutaraldehyde-treated tracheal matrix.

The macroscopic observations of CAMs treated with decellularized and genipin-treated tracheal matrices showed that all samples were surrounded by allantoic vessels that developed radially toward the implant in a spoke-wheel pattern (Fig. 3A–D). New vessels were occasionally arranged in loops around the samples, suggesting that the rabbit tracheal decellularized and genipin-treated matrices could positively affect the growth and organization of the network of CAM vessels. The effect of the tracheal matrices on direct blood vessel growth was quantified as the total number of converging blood vessels (Fig. 3E). All of the evaluated decellularized and genipin-treated tracheal matrixs samples induced a significant (p < 0.05) increase compared to that induced by Gelfoam (used as negative control) and glutaraldehyde-treated tracheal matrices.

### *in vivo* biocompatibility properties

**Figure 4:**
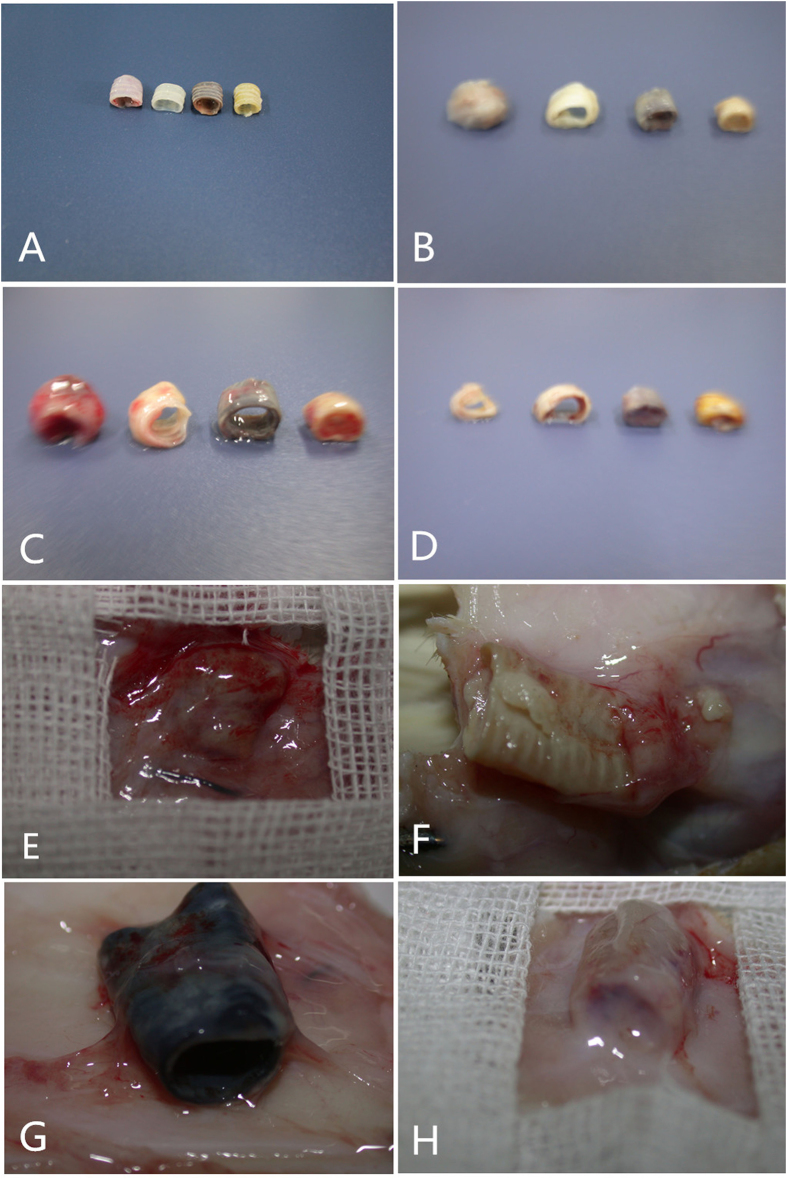
Macroscopic appearance of an explanted scaffold. The native trachea, decellularized trachea, genipin-crosslinked decellularized trachea, and glutaraldehyde-crosslinked decellularized trachea matrices after being implanted in rats for 0 (**A**), 7 (**B**), 15 (**C**), and 30 (**D**) days. The native trachea (**E**) and glutaraldehyde-crosslinked decellularized trachea matrices (**H**) exhibited a thick fibrous capsule with a large amount of pus and a damaged trachea 30 days after implantation. The optimal decellularized trachea (**F**) and genipin-crosslinked decellularizedtracheae (**G**) were covered by a thin capsule and neovascularized on the surface 30 days after implantation.

**Figure 5:**
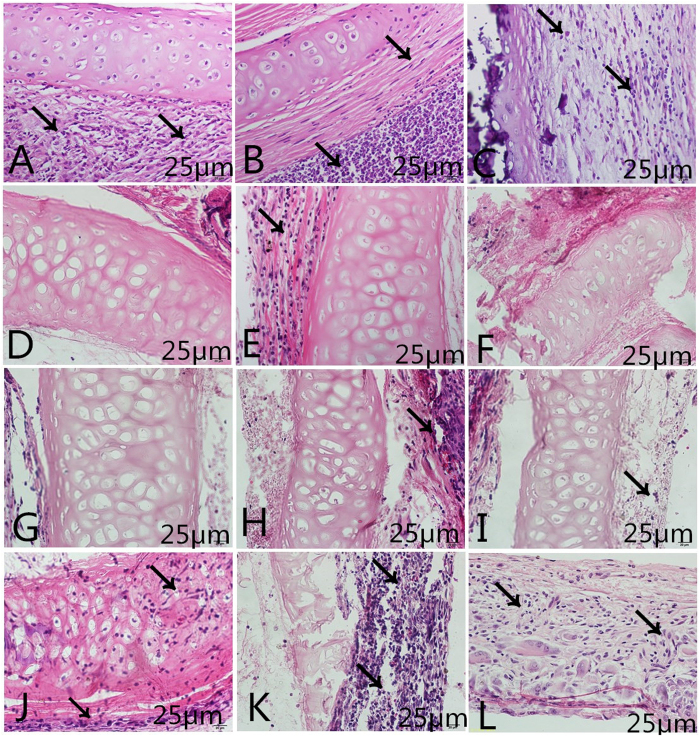
Xenotransplantation in SD rats. Transverse sections of native trachea (**A**–**C**), decellularized trachea (**D**–**F**), genipin-crosslinked decellularized trachea (**G**–**I**), and glutaraldehyde-crosslinked decellularized trachea matrices (**J**–**L**) after being implanted in rats for 7 (**A**,**D**,**G**,**J**), 15 (**B**,**E**,**H**,**K**) and 30 (**C**,**F**,**I**,**L**) days. The tissue sections are stained with (**H**,**E**). The black arrow indicates inflammatory cells. The scale bars are 25 μm.

**Figure 6:**
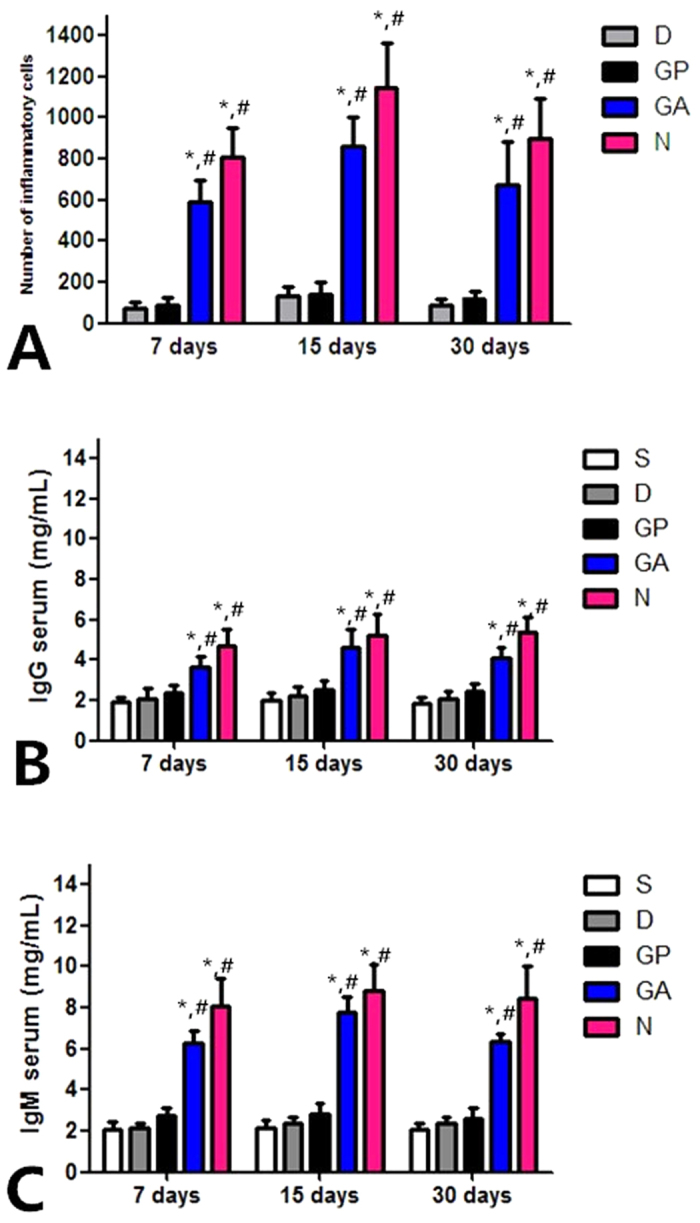
(**A**)Densities of inflammatory cells observed for the xenotransplantation matrices. (**B**,**C**)*In vivo* evaluation of IgG and IgM levels in recipient rats. S,Sham; (**D**), decellularized trachea; GP,genipin-crosslinked decellularized trachea; GA,glutaraldehyde-crosslinked decellularized trachea; N, native trachea; *P < 0.05 compared with decellularized trachea group at the same time point; ^#^P < 0.05 compared with genipin-crosslinked decellularized trachea group at the same time point.

The surgical operation and matrix implantation were well tolerated in all animals, with the rats of groups A/C/D surviving xeno-implantation with no sign of wound infection, inflammation or health impairment. In xenotransplantation models, macroscopic inspection showed that the decellularized matrices and genipin-treated tracheal matrices were resistant to collapse *in vivo* after 7, 15 and 30 days (Fig. 4A–H). Compared to the native trachea (Fig. 5A–C) and glutaraldehyde-treated tracheal matrices (Fig. 5J–L), the decellularized matrices (Fig. 5D–F) and genipin treated tracheal matrices (Fig. 5G–I) displayed no signs of biological incompatibility in terms of hyperacute (Fig. 5A,D,G,J), acute (Fig. 5B,E,H,K), or chronic (Fig. 5C,F,I,L) rejection, inflammatory cell response, or health impairment during the study period. After 7, 15, and 30 days of implantation, the decellularized matrices and genipin treated tracheal matrices showed a significantly lower inflammatory reaction compared to that observed with control tracheae (Fig. 6A), and no detectable increases in the IgM or IgG levels were observed in recipient rats (Fig. 6B,C).

## Discussion

Various natural materials have been applied to trachea tissue engineering, including decellularized tissue, collagen, hyaluronic-acid, and silk fibroin^15^. Decellularized tissue currently appears to be the most promising approach to obtaining suitable scaffolds to engineer a variety of tissues and organs^16^. Various detergents and solutions and physical techniques used to obtain an acellular scaffoldare available^17^. Given that the extracellular matrix (ECM) is important for tissue regeneration, cell homing, and differentiation, one must consider the disruption of the ECM while selecting the most suitable tissue decellularization process. To date, the only clinically acceptable method for producing a decellularized tracheal graft is a detergent enzymatic approach based on the use of deoxycholate and DNase^18^. This method provides a nearly acellular airway matrix of non-immunogenic and fairly native mechanical properties. Zang *et al.*^19^ confirmed that the decellularized tracheal matrix scaffold did not induce significant allograft rejection or foreign body reaction *in vivo*. Although the construct supported reepithelialization, stem cell-derived chondrocytes failed to engraft in the heterotopic environment and represent a focus of future investigations.

The detergent-enzymatic treatment was initially developed to isolate the basement membrane using sodium deoxycholate supplemented with distilled water and DNase. Ionic detergents, such as sodium deoxycholate, are effective in solubilizing cellular membranes, where major histocompatibility complex antigens reside. During the decellularization process, the extracellular matrix is disrupted, which allows the exposure of cells to decellularization agents and the removal of cellular material^20^. The process will affect the composition and structure of the extracellular matrix. Glycosaminoglycan is the main component of tracheal cartilage and, combined with its water storage capacity, provides the trachea with its mechanical strength to resist compressive forces. Glycosaminoglycan loss also explains the early reduction in compressive strength after decellularization^5^. Human tracheae were decellularized and stored for one year in PBS at 4 °C in the presence of antibiotics and anti-mycotics, and their structural, mechanical, and angiogenic properties were compared to baseline values. The results showed that the stored human decellularized tracheae were increasingly degraded, resulting in a loss of extracellular matrix architecture in particular of the collagenous and elastic fibre structure, and decreased mechanical and angiogenic properties^10^. Elliott *et al.*^21^ reported a case of a 12-year-old boy who was born with long-segment congenital tracheal stenosis and pulmonary sling. His airway had been maintained by metal stents, but after failure, a cadaveric donor tracheal scaffold was decellularized and used for a transplant. Eighteen months after surgery, he had a normal chest CT scan and ventilation-perfusion scan. The graft did not have biomechanical strength focally, so the patient had to retain a stent until 18 months^22^. To improve the mechanical properties of decellularized tracheae, genipin is a naturally derived cross-linking agent, that has been demonstrated to be sufficient to provide a scaffold that retains an adequate microstructure and mechanical response for tissue engineering applications^23^.

Multiple exogenous cross-linking approaches (chemical or physical) have been evaluated to find an ideal procedure to stabilize the mechanical integrity and natural compliance of collagen-based biomaterials^24^. Various synthetic cross-linking reagents, including formaldehyde, glutaraldehyde, dialdehyde and epoxycompound have been used on natural tissues; however, the application in clinics has been limited by their sideeffects, such as high cytotoxicity, mismatched mechanical properties and post-implantation calcification, which impairs their biocompatibility^12^. In contrast, genipin has recently been shown to be as effective as glutaraldehyde in improving the stability of collagen-based biomaterials, forming stable cross-linked products with less *in vitro* cytotoxicity (approximately 10,000 times less) and a lower *in vivo* inflammatoryresponse^25^,^26^,^27^. Thus, genipin appears to be a promising cross-linkin gagent for tissue engineering applications.

The purpose of cross-linking an acellular scaffold is to improve its stability and assure that the graft can persist *in vivo* without being subjected to host enzymatic degradation (e.g., collagenases and connective tissue proteases) before it can support itself. Genipin is completely naturally derived (it is obtained from geniposide, isolated from the fruits of gardeniajasminoides) and reacts spontaneously with amino acid chains or proteins, inducing an intramolecular and intermolecular cross-linking cyclicstructure within collagen fibres. The application of genipin has previously been used asa technique for articular cartilage repair^28^, to modulate the release of growth factors^29^, and to improve the mechanical properties of tissue-engineered scaffolds. Genipin has anti-inflammatory, anti-thrombotic and anti-oxidative activities and can suppress vascular and endothelial cell inflammatory activation^30^,^31^,^32^,^33^. Moreover, *in vitro* biocompatibility testing has demonstrated that genipin induces cellular proliferation, maintains the ability to support epithelial regeneration^25^, preserves the activity and function of endothelial cells^34^, and affect chondrocytes and osteoblast proliferation only at high dosages^35^. Reich *et al.*^36^ demonstrated that liquid chemical sterilization with genipin may be a potential alternative for treating biomaterial and tissue grafts. By virtue of its crosslinking, genipin may sterilize tissues while maintaining their mechanical integrity. Baiguera *et al.*^23^ demonstrated that genipin-treated decellularized tracheal matrices showed no toxicity to cells: the cells adhered, grew and proliferated extensively on both sides.

Our previous research demonstrated that 7 DEM cycles are optimal for obtaining suitable extracellular matrix integrity in a non-immunogenic rabbit tracheal substitute^13^. The transverse mechanical stability is also preserved in matrices treated in this manner. This research demonstrated that there was a decrease in the compression performance after decellularization (1.43 ± 0.12 MPa) compared to native (1.72 ± 0.15 MPa) tracheal matrices (P < 0.05). Thus, we have evaluated the use of genipin (at a 1% concentration) to cross-link decellularized rabbit tracheae and the biological response elicited. After the cross-linking treatment, the tracheal matrix structure remained intact, and the microstructures of the ECM fibres were largely preserved. An evaluation of the mechanical properties revealed that the longitudinal elastic modulus in genipin-treated tracheae (2.06 ± 0.18 MPa) was significantly higher than that of decellularized tissues (1.43 ± 0.12 MPa) (P < 0.05), suggesting that the crosslinking treatment could increase the response of the tracheal tissue to withstand forces experienced *in vivo*.

The results presented herein also demonstrate that genipin cross-linking (26 ± 3) did not alter the angiogenic properties of the tracheal matrices compared to decellularized tracheal matrices (22 ± 2). None of the investigated scaffolds produced any increase in the mortality rates of the chicken embryos, demonstrating that the cross-linked matrices were well accepted and did not induce any cytotoxic effects. Yao *et al.*^37^ compared the angiogenic effects of cross-linked matrices to VEGF-loaded matrices and demonstrated that the two types of matrices exhibited similar angiogenic potentials in both the CAM assay and animal model experiment. Our results confirmed that the genipin-crosslinking treatment (26 ± 3) positively impacted the angiogenic potential of biomaterials compared to the negative control group (6 ± 2) (P < 0.05). This effect is likely related to the modification of the presentation and conformation of ligands on the ECM surface, which affects the ligand-receptor interactions.

To evaluate the *in vivo* biocompatibility of the cross-linking treatment, we implanted the tissues into unmatched rats, thereby mimicking xenotransplantation. The implants were left for 7, 15, and 30 days without immunosuppression. Histological evaluation of the implanted glutaraldehyde cross-linked matrices and native matrices showed a higher inflammatory reaction with respect to genipin-fixed tissues and decellularized tracheae. The genipin cross-linked tissues and decellularized tracheae displayed no histologic signs of local or graft rejection and a significantly reduced inflammatory reaction when compared to untreated tracheae, where as the IgG and IgM levels were comparable to those obtained for the sham control.

Tissue-engineered scaffolds also play a main role in providing not only three-dimensional physical support but also a microenvironment that can guide tissue regeneration until the host’s own tissue can support itself. Thus, suitable tissue regeneration requires an equilibrium between matrix degradation and host matrix formation^12^. The modification induced by a crosslinking treatment, which stabilizes the triple-helix structure of collagen, masks collagen cleavage sites and physically hinders the penetration of enzymes into biological tissue, could affect the rate and extent of enzymatic *in vivo* tissue degradation, leading to a less constructive remodelling response^38^.

## Conclusions

The data reported here suggest that genipin cross-linking improves the mechanical properties, both in transverse tensile and longitudinal compression performance, without altering the pro-angiogenic properties of the decellularized airway matrix or eliciting an *in vivo* inflammatory response.

## Additional Information

**How to cite this article**: Sun, F. *et al.* Genipin cross-linked decellularized tracheal tubular matrix for tracheal tissue engineering applications. *Sci. Rep.* **6**, 24429; doi: 10.1038/srep24429 (2016).

## Figures and Tables

**Table 1:** Transverse and longitudinal mechanical characteristics of native vs. bioengineered tracheae.

Mechanical	Native	Decellularized	Genipin	Glutaraldehyde
Transverse				
Max. force (N)	9.62 ± 0.81	8.13 ± 0.49	11.19 ± 0.93*	10.83 ± 0.92*
Tensile testing (MPa)	4.62 ± 0.57	3.57 ± 0.23	5.51 ± 0.65*	5.09 ± 0.20*
Longitudinal				
Compression testing (MPa)	1.72 ± 0.15*	1.43 ± 0.12	2.06 ± 0.18*	2.11 ± 0.21*

DEM, detergent-enzymatic method; *P < 0.05 compared with decellularized trachea; N, newtons; Max. force, maximum applied force.
